# POPULATION ATTRIBUTABLE FRACTION OF POOR ORAL HEALTH FOR THE INCIDENCE OF DEMENTIA: A POPULATION-BASED COHORT STUDY

**DOI:** 10.1093/geroni/igad104.0050

**Published:** 2023-12-21

**Authors:** Xiang Qi, Bei Wu

**Affiliations:** New York University, New York City, New York, United States; New York University, New York City, New York, United States

## Abstract

Emerging evidence indicates a positive association between suboptimal oral health and increased risks of dementia. Despite the preventable and treatable nature of many oral health problems, oral health has not been considered in the risk factors for dementia that shape public health policy and research priorities. We aimed to quantify and compare the fractions of incident dementia attributable to poor oral health and other modifiable risk factors. The research included a nationally-representative sample of 15,474 cognitively intact adults aged≥50 from the Health and Retirement Study, with the outcome of incident dementia between 2006 to 2016. We estimated the multivariate-adjusted hazard ratio (HR) and population attributable fraction (PAF) of edentulism and nine risk additional dementia factors. Additional analyses were conducted on 1,239 individuals who completed the “Dental Health Module” in 2008, examining oral health problems as an exposure. Edentulism was associated with a higher risk of dementia for both men (HR,1.38; 95%CI, 1.15-1.66) and women (HR, 1.32; 95%CI, 1.16-1.51). The PAF of edentulism for dementia was 4.64%, which exceeded that of hearing impairment (3.58%) and hypertension (2.90%). Oral health problems were also a significant factor for men (HR, 2.01; 95%CI, 1.49-3.39) and women (HR, 1.15; 95% CI, 1.02-1.30), with a PAF of 11.84% among men, the second-largest following less education (22.60%). The substantial estimated proportion of dementia attributed to poor oral health in the aging population, particularly in men, warrants more attention. Dental care should be an integral component of a comprehensive approach to promoting cognitive health.

